# Unequal chances: ex ante fairness and individual control

**DOI:** 10.1038/s41598-020-78335-w

**Published:** 2020-12-14

**Authors:** Leticia Micheli, Nickolas Gagnon

**Affiliations:** 1 Institute of Psychology, Leibniz University Hannover, Hannover, 30159 Germany; 2 Department of Strategy and Innovation, Institute for Markets and Strategy, Vienna University of Economics and Business, Vienna, 1200 Austria

**Keywords:** Psychology, Human behaviour

## Abstract

Unequal financial outcomes often originate from unequal chances. Yet, compared to outcomes, little is known about how individuals perceive unequal distributions of chances. We investigate empirically the role of different sources of unequal chances in shaping inequality perceptions. Importantly, we do so from an ex ante perspective—i.e., before the chances are realized—which has rarely been explored. In an online survey, we asked uninvolved respondents to evaluate ex ante the fairness of unequal allocations of chances. We varied the source of inequality of chances, using a comprehensive range of factors which resemble several real world situations. Respondents also evaluated how much control individuals hold over the distribution of chances. Results show that different sources generate different ex ante perception of fairness. That is, unequal chances based on socioeconomic and biological factors, such as gender, family income and ethnicity, are evaluated to be unfair relative to the same chances based on effort, knowledge, and benevolence. Results also show that, for most individuals, there is a positive correlation between perceived control of a factor and fairness of unequal chances based on that factor. Luck appears to be an exception to this correlation, ranking as high in fairness as effort, knowledge, and benevolence, but similarly low in individual control as ethnicity, family income, and gender.

## Introduction

Unequal chances abound in the pursuit of financial rewards; erecting the landscape of the financial outcomes likely to be achieved by different groups of individuals. A striking recent study^1^ reports that in the United States “controlling for parental income, black boys have lower incomes in adulthood than white boys in 99% of Census tracts.” Policies such as additional investments for low-income children, diversity quotas in firms, and Affirmative Action in university admissions strive to redress these uneven chances before they translate into unequal outcomes. They attempt to “level the playing field” as the saying goes. Such policies can be observed across the globe. Brazil invests additional funds in the education of low-income children, through its Bolsa Família cash-transfer program. Norway as well as other European countries require a gender quota on corporate boards. Universities in India, South Africa, and the United States increase the admission chances of disadvantaged groups through Affirmative Action. Nevertheless, our understanding of how individuals perceive the unequal chances that these policies address is minimal. In fact, there is a noticeable empirical gap in the existing literature on distributive fairness ideals: we have limited knowledge about which types of unequal chances are considered to be fair from the ex ante standpoint—i.e., before chances are realized and incomes are known. We employ ourselves to bridge this gap.

Using a survey, we investigate the fairness perceptions of different factors used to distribute unequal chances, from the ex ante perspective. As many studies before^2–6^, we examine the fairness perceptions of an impartial or uninvolved spectator. While non-monetarily incentivized, the perspective of the spectator is of value because it is expected to be devoid of self-interest^3^. That is, the spectator’s judgment should be impartial, providing us with a normative account of what is considered fair in the ex ante domain.

We use a large set of factors, which include factors often serving as bases for unequal chances in society such as effort, knowledge, luck, gender, ethnicity, and family income. Those factors are more comprehensive than those that the literature on fairness has employed, enabling a more fine-grained exploration of differences among a range of situations that can generate unequal chances. The motivation for employing this wider range is twofold. First, our primary motivation is empirical, as whether differences exist in fairness perceptions is unknown for almost all chance factors studied here, and several of those unstudied factors such as ethnicity and gender are valuable for public policy. The only study investigating chance factors from an ex ante viewpoint^7^ also makes conclusions about “sheer luck” and “performance” by studying height and a mix of effort and knowledge, but those are proxies of unknown quality. Moreover, we add identification numbers (ID) to our factors because experimenters sometimes allocate students to treatments based on IDs in laboratory experiments to mimic random assignment, so that it is practical to understand the fairness of such a chance assignment. Second, from a theoretical viewpoint, liberal egalitarianism, which we discuss below, as well as a few other concepts could affect fairness perceptions of some of our factors. Those include repugnance^8^, psychological cost^9^, and indirect reciprocity^10^, which we discuss in the Methods section.

Moreover, we examine the perception of individual control over these factors and the relationship between control and ex ante fairness. This is important because economic and philosophy research on the liberal egalitarian fairness ideal has emphasized the positive relationship between factors perceived as within one’s control and fairness perception of different types of outcome inequality and the lasting theoretical difficulties in pinpointing exactly which factors are under one’s control^11–15^. Furthermore, whether this relationship extends empirically to chance inequality and ex-ante fairness judgements has not been explored. Lastly, we touch upon Affirmative Action, which raises the odds for disadvantaged groups over advantaged groups. We do this by testing whether it is fairer to provide higher chances to groups that are generally disadvantaged than to groups that are generally advantaged. Our hope is that understanding how individuals perceive unequal chances originating from different factors can provide insights to policymakers in terms of which unequal chances are perceived as justifiable and which may call for rectification.

The economic literature investigating inequality and redistribution decisions draws a line between ex post and ex ante fairness. While *ex post fairness* concerns the distribution of incomes between individuals once these incomes are known^16^, *ex ante fairness* applies to the distribution of chances that are to generate incomes^17^. The literature in psychology and management^18–21^ also studies the factor generating outcomes separately from the outcomes themselves and uncovers crucial elements that influence individuals’ appraisal of factors, such as respect, an opportunity to be involved or voice their concerns, and the absence of biases.

The empirical literature on redistribution focuses mostly on ex post fairness, once incomes are already known. Studies show that a substantial proportion of individuals display concern for equality of incomes, exhibiting a willingness to decrease their own income to improve the conditions of others through redistribution^4,6,22–26^. Studies have also examined ex-post fairness from the non-monetarily incentivized spectator viewpoint, showing that many individuals also prefer to equalize earnings of equally-deserving participants^4,6,27^ and judge such unequal situations to be unfair when asked in a hypothetical manner^3,5^.

There is also empirical evidence that individuals care about ex ante inequality, although the topic is less studied. For instance, recent economic experiments^28,29^ presented participants with unequal chances to win a lottery, and the opportunity to redistribute chances before the realization of that lottery (note that other types of experiments exist in the same vein^30^). In those experiments, many individuals indeed show concern for equality when redistributing chances, even if it reduces their own chances of winning. Similarly, experiments show that individuals do consider the distribution of initial chances in their redistribution decisions even after the realization of these chances, both when they are themselves involved^31–33^ or when spectators^2,33^. Finally, theoretical work on social preferences for obtaining a fair chance has been the focus of a number of papers in the past twenty years^31,34–37^.

Studies investigate the fairness perception and the distribution decisions of incomes that have been generated through different factors, but they do so almost exclusively from an ex post perspective. For example, they examine whether the patterns of fairness perceptions and income distribution decisions differ if income inequality is caused by different factors, such as effort, knowledge, and luck^4–6,27,38^. Typically, a sizeable proportion of individuals are more accepting of inequality caused by effort and knowledge than luck, both when involved^4,6,27,38^ and when spectators^4,6,27^. The prominent explanation for this type of behavior is the view that individuals should be held accountable for factors under their control, such as effort and knowledge, but not for others which they cannot influence, such as luck. This is the position taken by the liberal egalitarian fairness ideal^12^ and the accountability principle^6^. Several earlier equality concepts in philosophy also hold individuals responsible for their choices, the difference often being to which extent one is responsible for certain factors and not for others, a notoriously difficult matter to determine theoretically^13–15^.

In contrast, how different factors behind the distribution of chances affect the ex ante fairness perception of these chances has scarcely been explored by empirical studies. We believe that this missing step is socially valuable because many policies—such as the Affirmative Action described in the first paragraph—intend not to correct incomes once unequal chances have been realized, but to correct different types of unequal chances before they are realized. Furthermore, whether the positive relationship between perceived control and fairness of inequality documented ex post is typical from an ex ante perspective has not been researched much. Studying ex ante perceptions is essential because our knowledge regarding ex post fairness views does not necessarily inform us about ex ante fairness views. For instance, theoretically, a society’s fairness evaluation of a factor based on luck may differ ex ante and ex post: providing equal chances of obtaining either a high or low income to everyone might be judged as fair at the outset, only to be considered unfair once unequal incomes have been randomly allocated^39^.

The only study that has empirically investigated the role of different factors in ex ante fairness views aims to compare how distributions of chances based on “sheer luck” vs. “performance” impact individuals’ ex ante choice of future redistribution by employing chances based on height vs. a combination of effort and knowledge in a quiz^7^. Using those measures of luck and performance, the study finds that, ex ante, individuals are more prone to support future redistribution when luck influences chances than when performance does, which provides some evidence that liberal egalitarianism is present ex ante. We add to this research by investigating fairness perceptions of a wide range of factors underlying unequal distribution of chances, which are mostly chosen to resemble real-world situations of inequality. Interestingly, we measure fairness perceptions of height and luck, and find large differences in fairness ratings between the two, whereas luck rates similarly in fairness as effort and knowledge. Moreover, we measure individuals’ perceptions of control over the different factors. We note that the previous study also included questions regarding fairness perceptions of factors, but did not find significant differences between the reported fairness of the height and the effort and knowledge factors. However, as the author reports, its use of a between-subject design might generate too much noise to distinguish fairness differences between the factors. Our within-subject design is likely to alleviate this issue.

Finally, we note the following point about our measures of fairness. In economic studies of distributive moral ideals, it is theoretically assumed that individuals hold fairness preferences or ideals that translate into income redistribution decisions, and the common empirical way of studying fairness is then through revealed preferences: we measure redistribution decisions and then infer preferences from those decisions^4,6,7^. We instead use survey questions about fairness perceptions in a hypothetical scenario, as others have done^3,5,40^. Note that economic experiments in other domains also sometimes employ non-incentivized measures of fairness perceptions, e.g., in labor experiments^41,42^.

## Methods

We recruited 82 undergraduate students from the online recruitment system for economic experiments (ORSEE)^43^ of the Behavioral and Experimental Economics Laboratory (BEELab) at the School of Business and Economics of Maastricht University (The Netherlands) to take part in an online Qualtrics survey. We obtained informed consent from participants to take part in the study. They completed the survey in April and May 2017 and received 5 EUR for their participation. In terms of demographic characteristics, 41% are men and the mean age is 21.5 years. The study was conducted following the peer-approved factors established by Maastricht University’s Behavioral and Experimental Economics Laboratory (BEELab). Our study was approved by the Maastricht University’s Behavioral and Experimental Economics Laboratory at a public ethics review and project proposal meeting that is mandatory for all scholars who wish to use the BEElab facilities. All methods were performed in accordance with the relevant guidelines and regulations of the BEELab and Maastricht University for studies with human participants.

Participants considered a hypothetical scenario in which unequal chances to win a prize of 10 EUR are allocated between two other individuals. Participants could not win the prize themselves, and, for simplicity, we framed chances as lottery tickets. Chances are always allocated in the following manner: one individual has a $90\%$ chance, and the other has a $10\%$ chance. Only one of the two individuals wins the prize. Participants were asked questions concerning different factors determining who receives which chance. The scenario was described to participants in the following manner:

*Consider a situation in which lottery tickets are distributed between two persons. Generally we ask you to assume that these two persons have a similar social, economic and educational background. In some cases this will not be so and you will be explicitly informed about it. Each ticket gives a* $10\%$ *chance to win the lottery. There can be only one winner in the lottery, who receives 10 EUR as a prize. The ticket allocation is as follows: one person gets 9 tickets and thus has a* $90\%$ *chance of winning the lottery prize, whereas the other person gets 1 ticket and thus has a* $10\%$ *chance of winning the lottery prize. The allocation of tickets is based on various factors.*

We then presented procedures varying 11 different factors in random order. Although all procedures presented led to the same consequences (i.e., one person receiving a $10\%$ chance to win a single lottery prize and the other receiving a $90\%$ chance of winning that same prize), these procedures differed with respect to the factor that determines who receives which chances. The factor presented was one of the following: benevolence, knowledge, effort, luck, student identification number (ID), height, weight, nationality, gender, ethnicity (skin color), and family (parental) income. We set a highly unequal distribution of chance $90\%$ vs. $10\%$) to make chance inequality more salient than outcome inequality. This distribution is similar to what has been used previously^31^.

All factors were presented once in our “Regular” Block, where the higher chance was allocated to individuals who were benevolent, more knowledgeable, exerted more effort, lucky, with a higher ID, tall, heavy, man, German, light-skinned, or from a high-income family. We also presented participants with the same factors in our “Reverse” Block, this time allocating higher chances to the opposite individuals (e.g., individuals who are non-benevolent, dark-skinned, women or from a low-income family). The order of blocks was randomized. Except for a few pre-selected factors, we are solely interested in the “Regular” Block because the factors in this block described cases that generally occur more often in real life, such as advantaging men, light-skinned individuals, or individuals from a high-income family. All factors are presented in the “Reverse” block simply to avoid disclosing the factors we wanted to focus on in this block. Those pre-selected factors of interest in the “Reverse” block are chosen to evaluate whether asymmetries in fairness might exist for them, which we explain below.

Table 1 summarizes the criteria for receiving higher chances, for each factor of each block. For benevolence, we presented the case of an individual who decided to help a person in need and another individual who decided to *not* help a person in need. In this specific case, higher chances are allocated to the benevolent person in the “Regular” Block, while higher chances are allocated to the non-benevolent person in the “Reverse” Block. For knowledge, we used a general knowledge quiz, where the person with a higher number of correct answers would receive higher chances in the “Regular” Block and lower chances in the “Reverse” Block. For effort we employed a slider task^44^, a common effort task in experimental economics, which is explained in the example below. Again, the person exerting more effort would receive higher chances in the “Regular” Block and lower chances in the “Reverse” Block.

We make a few observations about this effort task. First, we think that the effort scenario that we present makes it clear that the two individuals in the scenario are aware that their effort determines their chances of winning the prize, although it does not explicitly specify the beliefs of individuals in the scenario. Control ratings by respondents in the Results section are in line with this, suggesting participants understand that chances depend on individuals’ effort. Second, we cannot rule out that performance in the slider task is fully unrelated to ability, but it is a gruesome manual task that relies on the willingness to put in effort. Third, since it is one of the most common tasks used in economic experiments, participants in our laboratory pool are likely to be familiar with this task by having experienced it themselves. Even for those who have not, the task and its mechanical nature are relatively easy to comprehend in the description that we provide.

Then, for luck we adopted a 6-side die roll where one person would roll a number equal or smaller than 3 and receive higher chances, whereas the other would receive lower chances. For the ID factor, we used greater and smaller student ID numbers (which are generally allocated by order of enrolment, although this might be unknown to students). For height we contrasted taller and shorter individuals, while for weight we contrasted heavy and light persons and for gender we employed women versus men. For nationality, we made use of Germans versus non-Germans (German is the most common nationality in the participants pool). For ethnicity, we contrasted light-skinned to dark-skinned individuals. Finally, for family income we considered individuals coming from higher or lower income families.

Participants first rated the fairness of all factors for both the Regular and Reverse Blocks. After that, participants were exposed a second time to both blocks, again in random order, and rated the same factors in terms of how much individual control one has over chances received. Responses were given on a 7-point Likert scale ranging from *Very Unfair* to *Very Fair* and from *No Control* to *Full Control*. At the end of the study, participants provided demographic information, such as age, gender and parental income as well as other characteristics of themselves such as height and weight. The full instructions are provided in the Supplementary materials. As in many studies on moral ideals before us^2–6,27^, our participants’ answers do not affect their own financial welfare. We rely on participants’ intrinsic motivation to pay attention. Still, we made sure that our study provides adequate conditions for them to pay attention: all instructions are carefully explained, the study is designed to be short ($<20$ minutes) and participants are provided with messages warning them prior to a change in blocks or a change in ratings (i.e., fairness to control). The following two examples regarding effort and gender describe how the factors were presented to participants. Both examples belong to the Regular Block.

Effort Example: *Chances are allocated based on the results of a task that depends on effort. In the task, people see dots appearing randomly on horizontal bars on their computer screen. Their task is to position as many dots as possible in the middle of the horizontal bars. The person who places more dots in the correct position in a given time receives a* $90\%$ *chance to win, and the person who places less dots in the correct position receives a* $10\%$ *chance to win.*

Gender Example: *Chances are allocated based on gender. The two persons have different genders. The man receives a* $90\%$ *chance to win, and the woman receives a* $10\%$ *chance to win.*

**Table 1:** Criteria for receiving higher chances in the Regular and Reverse blocks.

Factor	Regular block	Reverse block
Benevolence	Choosing to *help* someone in need	Choosing *not to help* someone in need
Knowledge	*Higher* results in a knowledge quiz	*Lower* results in a knowledge quiz
Effort	Correctly position *more* dots in the task	Correctly position *less* dots in the task
Luck	Roll of 6-faced die gives *3 or less*	–
ID	*Higher* student ID number	*Lower* student ID number
Height	*Taller* person	*Shorter* person
Weight	*Heavier* person	*Lighter* person
Gender	*Man*	*Women*
Nationality	*German*	*Non-German*
Ethnicity	*Lighter* skin color	*Darker* skin color
Family Income	*Higher* parental income	*Lower* parental income

*Notes:* We did not reverse Luck because this would simply mean changing the winning numbers of the die roll, which should have no effect on its fairness and control ratings.

**Hypotheses:** We test whether there are differences in fairness among factors, and whether fairness is a correlate of the perception of control. Our first hypothesis is that there are differences in fairness perceptions of chance factors. If the liberal egalitarian ideal previously documented in environments without chances^4–6,38^ translate to chance factors, effort and perhaps knowledge will be evaluated as fairer than other factors. In addition, several other concepts could be at play. For instance, low chances based on morally hard-to-justify factors such as ethnicity, gender, and income could create strong repugnance^8^, which would lower fairness perceptions. Understanding the extent of the psychological cost of ethnic and gender discrimination on those receiving them^9^ could also decrease the perceived fairness of chance based on those factors, and indirect reciprocity^10^ could increase the fairness of high chances based on benevolence or good deeds.

Our second hypothesis is that factors with low individual control are perceived as more unfair, in line with liberal egalitarianism^12^. Furthermore, we test for asymmetries in fairness ratings when chances favor one group over the other for the following factors: benevolence, knowledge, effort, gender, ethnicity, and family income. We expect that asymmetries in these cases will arise for two reasons. First, previous research shows that individuals reward “merit” in the case of effort^4,6,11,27^ and knowledge^38^, and this might extend to benevolence. This can lead to individuals finding it fairer to allocate higher chances to those who were benevolent, who provided more correct answers in the knowledge quiz and who exerted more effort than to those with opposite characteristics. Second, we conjecture that one might find it fairer to favor generally disadvantaged groups because this compensates existing inequality. Hence, our third hypothesis is that participants rate factors as fairer when women, individuals with dark skin and low parental income receive higher chances than men and individuals with white skin and high parental income.

## Results

**Result 1—ex ante fairness ratings:** *Regular-block factors advantaging individuals who are benevolent, knowledgeable, exert effort and are lucky, score high on fairness, whereas factors advantaging individuals based on socioeconomic or biological factors (e.g., gender, height, skin color or parental income) score low on fairness.*

Table 2 presents the mean fairness ratings of Regular-block factors. We use non-parametric Wilcoxon signed-rank tests to analyze pairwise differences between factors in their distribution of fairness ratings (N=82). We employ such a non-parametric rank-based technique because the fairness data that we have is measured on an ordered (non-metric) scale and heavily censored below (49.6% of answers indicate the minimum fairness level). Stars in a cell indicate that the factor in the column is fairer than the corresponding row. *P*-values are corrected for multiple comparisons using the Holm-Bonferroni method^45^ and are indicated by the number of stars. For completeness, fairness ratings of the factors in the Reverse Block can be found in the Supplementary materials.

This result indicates that not all chance inequalities are considered equally fair from the ex ante viewpoint, which is in line with what other studies have found for the ex post perspective. Indeed, despite the fact that all factors led to an unequal distribution of chances between individuals, some factors were considered fairer than others. Unlike for the ex post perspective, however, factors giving higher chances to those who exert effort or are knowledgeable are not significantly fairer than luck, which simply randomly allocates chances. Factors based on socioeconomic and biological criteria are the ones which really differ from luck, by being substantially less fair.

Although we do not aim to formalize groups in terms of fairness, we can assay their presence by conducting a Principal Component Analysis (PCA). It detects three main components (eigenvalues $\ge$ 1), which we interpret as groups, in terms of fairness: (1) height, weight, gender, nationality, ethnicity, and family income (all biological and sociological factors that we use; 33.9% of variance), (2) effort, knowledge, and benevolent action (19.0% of variance), and (3) luck and ID (9.5% of variance). Given that the last group is only marginally detected (eigenvalue = 1.04) and explains little of the variation, the picture arising from this analysis is generally similar to what we obtained by engaging in pairwise comparisons of factors.

We also examine whether a participant’s characteristic could influence their fairness ratings when facing a situation that allocates chances based on that specific characteristic, e.g., whether a man rates unequal chances advantaging a man any differently than a woman does. To do this, we conduct a non-parametric Wilcoxon-Mann-Whitney test (or Wilcoxon rank-sum test) to test whether this is the case for each of the following 7 self-reported characteristics: gender, height (below or above median sample value), weight (below or above median sample value), skin color, general knowledge (below or above median sample value), laziness (below or above median sample value), and parental income. We find no statistically significant effect for each of the 7 tests on fairness ratings (*p*-values > .159). This is line with the advantage of using spectator participants: their judgement is as impartial as possible as they have no stake in the situation.

Overall, our first result suggests that individuals may be prone to accept, ex ante, certain inequality of chances, e.g., when they come through effort, knowledge, or even luck, while endorsing policies that rectify others, e.g., when they rest on biological and socioeconomic factors. Next, we test whether individuals’ judgments of ex ante fairness differ depending on who is advantaged by the unequal distribution of chances.

**Table 2:** Mean fairness and Wilcoxon signed-rank test comparisons.

Factor	Benevolence	Effort	Luck	Knowledge	ID	Height	Weight	Nationality	Family Income	Gender	Ethnicity
Benevolence	5.38 (1.47)										
Effort		5.04 (1.53)									
Luck	*		4.63 (1.77)								
Knowledge	***	**		4.38 (1.88)							
ID	***	***	***	***	2.17 (1.67)						
Height	***	***	***	***	**	1.37 (.75)					
Weight	***	***	***	***	**		1.35 (.60)				
Nationality	***	***	***	***	***			1.33 (.85)			
Family Income	***	***	***	***	***				1.29 (.60)		
Gender	***	***	***	***	***					1.21 (.46)	
Ethnicity	***	***	***	***	***	**	**				1.13 (.34)

*Notes:* Standard deviations are indicated in parentheses. Stars in a cell indicate that the factor in the column is fairer than the factor in the corresponding row. Holm-Bonferroni-corrected *p*-values: * ${p<.05}$; ** ${p<.01}$; *** ${p<.001}$.

**Result 2—asymmetries in ex ante fairness ratings:** *It is fairer to advantage individuals who are benevolent, dark-skinned, knowledgeable, and exert more effort, compared to advantaging individuals with the opposite characteristics. It is not fairer to advantage women over men.*

In five of the six cases of interest (benevolence, effort, knowledge, family income, gender, skin color), as hypothesized, the ex ante fairness rating differs depending on the individual who is favored with higher chances. The exception is gender, for which, unlike what we hypothesized, we did not find a significant asymmetry. Table 3 reports the mean fairness for the factors advantaging one group (Regular Block) versus the other group (Reverse Block). Non-parametric Wilcoxon signed-rank tests (N=82) are used to compare fairness ratings of a factor in the Regular Block vs. the Reverse Block, separately for each of the six factors (e.g., favoring someone who was benevolent vs. someone who was not benevolent, then favoring a man vs. favoring a woman). Stars in the table indicate whether favoring one type of individuals was significantly fairer than favoring the other type for each factor separately. The asymmetries that we identify can occur for different reasons. First, asymmetries in fairness in factors that involve some sort of merit (benevolence, effort, knowledge) can occur because individuals find it fair to distribute chances in a meritocratic manner. Second, individuals may consider it fairer to give higher chances to disadvantaged groups similar to what Affirmative Action policies often do.

We make two observations. First, finding no asymmetries in the case of gender might be due to the specific setting that we study—a Dutch business school with a large share of women among its student body, which for students is likely not highly unequal in terms of gender. Second, although we do find asymmetries in fairness, factors that favor disadvantaged groups are still generally considered unfair compared to factors that advantage those who are benevolent, exert effort, or are knowledgeable. This result reflects to some extent the mixed support affirmative action can receive. Even though individuals recognize that giving more chances to the disadvantaged is fairer, such factors are still considered relatively unfair. Alternatively, the low relative fairness of the affirmative action factors that we use might be due to the somewhat egalitarian environment of the students. Another reason for this result is that these factors create a large amount of inequality by distributing very unequal chances instead of slightly advantaging the generally-disadvantaged group.

**Result 3—Control and fairness:** *A majority of individuals consider factors that one has control over to be fairer. Interestingly, luck stands out as the exception: it ranks high in fairness but low in control.*

Table 4 details the mean control ratings of the Regular factors. Since our fairness and control data are both measured on an ordered scale, we calculate the non-parametric Spearman correlation between fairness and control for each individual (N=79), for the 11 factors contained in the Regular Block. Note that we cannot calculate the correlation for 3 participants because there are no variations in their fairness or control answers. For the 79 remaining participants, the Spearman $\rho$ is positive for 95% of them, at least of medium strength ($\rho$ $\ge$ .30) for 92%, and strong ($\rho$ $\ge$ .60) for 63%. The $\rho$ is significantly greater than zero at the .05 significance level for 62% of participants, and at the .10 level for 71%, while it is significantly smaller than zero at the .10 level for none. For the interested reader, the mean control ratings for the Reverse Block are shown in the Supplementary materials.

To provide a picture of the correlation at the group level, we can employ an individual-level random effects parametric Tobit regression with lower-bound censoring. Fairness is the dependent variable and control is the explanatory variable. The resulting control coefficient of 0.985 (SD=.048, N=902, *p* $<.001$) is significant and indicates that, on average, there is a positive correlation between control and fairness. Moreover, we note that a PCA reveals the presence of two main groups (eigenvalues $\ge$ 1) in terms of control: (1) height, gender, nationality, ethnicity, and income (all biological and sociological factors that we employ, except weight; 35.8% of variance), and (2) effort, knowledge, benevolence, and weight (22.3% of variance). This paints a picture similar to the one we obtain from pairwise comparisons of factors in terms of controls.

We also examine whether a participant’s characteristic could influence his or her control ratings when facing a situation that allocates chances based on that characteristic. We conduct a non-parametric Wilcoxon-Mann-Whitney test to test whether this is the case for each of the following 7 self-reported characteristics: gender, height (below or above median sample value), weight (below or above median sample value), skin color, general knowledge (below or above median sample value), laziness (below or above median sample value), and parental income. We find no statistically significant effect for each of the 7 tests on control ratings (*p*-values > .153).

Our third result indicates that a substantial share of participants exhibit a positive correlation between fairness and control when they make ex ante judgments about unequal distributions of chances. This is in line with what other studies have found for ex post judgments of unequal outcomes, for involved participants^4,6,27,38^ and for spectator participants^4,6,27^. That is, the judgments of many individuals appear broadly consistent with the liberal-egalitarian fairness ideal, whereby individuals find unequal chances based on factors over which one has control to be fair. However, the fact that the factor randomly allocating high chances is as fair ex ante as the factors advantaging those who exert effort or are knowledgeable—while at the same time being judged to be under no control—is inconsistent with the liberal-egalitarian fairness ideal.

**Table 3:** Asymmetries and Wilcoxon signed-rank test comparisons.

Higher chance to	Mean fairness	Difference
Benevolent	5.38 (1.47)	***
Non-benevolent	1.93 (1.44)	
More effort	5.04 (1.53)	***
Less effort	2.52 (1.65)	
More knowledgeable	4.38 (1.88)	***
Less knowledgeable	2.17 (1.39)	
High family income	1.29 (.60)	***
Low family income	2.74 (1.79)	
Man	1.21 (.46)	
Woman	1.32 (.83)	
Ethnicity—light skin	1.13 (.34)	**
Ethnicity—dark skin	1.44 (.98)	

Notes: Standard deviations are indicated in parentheses. Stars in a cell indicate a significant difference in fairness for a factor depending on who is advantaged. Holm-Bonferroni-corrected *p*-values: * ${p<.05}$; ** ${p<.01}$; *** ${p<.001}$.

**Table 4:** Mean control and Wilcoxon signed-rank test comparisons.

Factor	Benevolence	Effort	Knowledge	Weight	Luck	Family Income	ID	Gender	Nationality	Height	Ethnicity
Benevolence	5.91 (1.51)										
Effort	***	5.22 (1.41)									
Knowledge	***		4.98 (1.49)								
Weight	***	***	***	2.95 (1.65)							
Luck	***	***	***	***	1.55 (1.00)						
Family Income	***	***	***	***		1.50 (1.03)					
ID	***	***	***	***			1.45 (.72)				
Gender	***	***	***	***				1.28 (.76)			
Nationality	***	***	***	***					1.28 (.86)		
Height	***	***	***	***						1.27 (.74)	
Ethnicity	***	***	***	***							1.26 (.70)

Notes: Standard deviations are indicated in parentheses. Stars in a cell indicate that the factor in the column is under more control than the factor in the corresponding row. Holm-Bonferroni-corrected *p*-values: * ${p<.05}$; ** ${p<.01}$; *** ${p<.001}$.

## Discussion

This study addresses a gap in the existing distributive fairness ideals literature regarding how individuals evaluate the ex ante fairness of distributions of chances based on different factors. We exposed impartial or uninvolved spectators to different factors used to distribute unequal chances to win a monetary prize between two individuals. Before the chances were realized, participants were asked to rate the fairness of each factor as well as to rate how much individual control each factor allowed for.

Our results indicate that, as other studies have found for ex post inequality, for both involved participants^4,6,27,38^ and for spectator participants^4,6,27^, the fairness of ex ante inequality depends on the causes that generate this inequality. Factors advantaging those who are benevolent, knowledgeable, exert more effort or advantaging those who are simply lucky are rated similarly in terms of fairness by uninvolved spectators. The assessment of luck differs from the ex post perspective of both involved and spectator individuals, in which factors that advantage individuals based on luck are often considered to be less fair than meritocratic factors that advantage those who exert more effort or who are more knowledgeable^4,6,27,38^.

The group of factors based on gender, height, weight, nationality, ethnicity or family income, which we could group together as biological and socioeconomic factors for ease of exposition, are substantially less fair than the other factors that we study, none of which are biological or socioeconomic. The difference between those two types of factors is in line with what we regularly witness in societies: unequal chances based on biological or socioeconomic characteristics are denounced as unfair and can elicit strong reactions, such as social movements against racism and sexism demanding equal chances for members of different groups. Generally, the different fairness ratings that we observe reinforce the need to differentiate inequality and fairness^46^.

We note that the differences in fairness ratings between the different factors in our study appear unlikely to be due to individuals being more sensitive to some specific information (e.g., gender or ethnic information). If this was the case, one could expect individuals to be more sensitive to scenarios in our study in which their own attributes or characteristics served to create chance inequality. However, results show that individuals’ characteristics (e.g., gender, skin color, general knowledge, weight, parental income) are not significantly related to their fairness ratings or to their control ratings in situations where those characteristics determine chance inequality. In addition, despite keeping information on factors constant in the Regular and Reverse blocks (only changing which individual receives higher chances), we observe variability in the fairness ratings of factors between the two blocks. Finally, we note that the differences and ordering in control ratings between factors are not exactly the same as for fairness ratings (e.g., luck and weight). Still, it would be interesting to further explore whether and how sensitivity to information can impact fairness judgments in future research.

The asymmetry observed in the fairness of certain factors is consistent with at least a substantial number of individuals supporting Affirmative Action for disadvantaged groups, such as ethnic minorities. For instance, 63% of Americans reported in 2014 that they thought that affirmative action programs increasing black and minority university students are a “good thing”^47^, while 26% stated in 2019 that race should be a factor in university admission^48^. Even in our setting of a simple lottery among university students, it is indeed fairer to favor certain disadvantaged groups, such as individuals with dark skin. We observe, however, that increasing the chances of certain groups is still considered unfair on average in this context relative to advantaging those who exert more effort or are more knowledgeable. We do not find that it is fairer to give higher chances to one gender over the other, although we note that this might be the result of using students in a relatively gender-balanced environment. Overall, asymmetries in perceived fairness provide a potential source of expansion for current research.

Furthermore, factors allocating chances in a fairer manner rate higher in individual control. This is broadly consistent with the liberal-egalitarian fairness ideal that a large share of individuals have been shown to hold in terms of ex post fairness^4^. Interestingly, unlike ex post, liberal egalitarianism fails to hold ex ante in the case of luck, which is considered both fair and outside of one’s control. On the one hand, the factor randomly allocating higher chances is considered as fair as the factors advantaging those who exert effort or are knowledgeable. On the other hand, whereas factors based on effort or knowledge are judged to be under individuals’ control, luck is obviously considered to be under no control, as are factors based on biological or socioeconomic factors.

How is it possible for the random allocation to be fair from an ex ante perspective when previous work^4,6,27,38^ highlight that it is unfair ex post? Theoretical work suggests that individuals can be time-inconsistent when making ex ante and ex post fairness judgements^39,49^. For instance, they can first support policies that aim at ex ante chance equality, but lead to unequal incomes, and, once these unequal incomes are realized, they then support policies that aim at ex post income equality. This problem can arise when, at the moment of making an ex ante choice, individuals do not take into account possible ex post inequality. A recent working paper also reports behavior consistent with this phenomenon: individuals often make an ex ante fair choice before outcomes are realized, only to then switch to an ex post fair choice once outcomes are known^50^. A qualitative comparison between our fairness results in the ex ante domain and fairness results of other studies in the ex post domain^4,6,27,38^ also suggests that individuals may make different ex ante and ex post fairness judgements. However, our study cannot provide a direct test of this time-inconsistency explanation because it does not measure individuals’ perception of both ex ante and ex post fairness, and differences in designs and contexts with previous studies could also have contributed to the observed differences. It would be interesting to investigate not only if fairness ratings differ ex ante and ex post, but to explore if the extent of these differences depend on the source of inequality. An extrapolation of our results would suggest that while differences may be observed for luck, other factors such as effort and knowledge might be considered equally fair from an ex ante and ex point view.

In addition, we note that it can be puzzling that individuals evaluate chances allocated based on luck as fairer than chances allocated based on other factors that could be easily rationalized as coming from luck, such as height. We note that luck being perceived as fair ex ante and unfair ex post may provide an explanation for this phenomenon. That is, on the one hand, an allocation of chances based on height may be seen through ex post lenses, as the luck that could determine height has already been realized. On the other hand, an allocation of chances based on luck may be seen as still not realized—i.e., as part of a compound lottery—and therefore be perceived through ex ante lenses. Another possibility is that the exception of luck might have arisen through social conventions. That is, although die rolls cannot be controlled, they could be perceived as fair because individuals often voluntarily opt to use them to settle indivisible claims, whereas individuals rarely settle claims based on height.

Importantly, our results on fairness contrast in some respects with the only previous study that has empirically investigated the role of different factors in ex ante fairness views^7^. That paper^7^ aimed to compare how distributions of chances based on “sheer luck” vs. “performance” impact individuals’ ex ante choice of future redistribution by using chances based on height vs. a combination of effort and knowledge in a quiz. While we do not study redistribution decisions, our results suggest that there are large differences in fairness perceptions between height and luck, whereas luck rates similarly in fairness as effort and knowledge. Future research will need to address whether this difference between height and luck also translates into a large difference in ex ante redistribution decisions.

Our findings carry two potential implications for policymakers. First, the causes of unequal distributions of chances need to be carefully considered if one wants to make chance-equalizing policies that correspond to the fairness perceptions of individuals. Second, considered with the results of other studies, our study suggests that individuals exhibit time-inconsistency regarding the fairness of different redistribution policies. That is, they may support policies that grant equal chances, but create income inequalities, only to later switch their support to policies that aim at equal incomes. A way to avoid this reversal is perhaps to conscientiously consider the ex post inequalities that equal chances may generate, whenever there are public debates on equal-chance versus equal-income policies.

We also note that in our study we opted to investigate the judgments of uninvolved spectators, whose judgment should be impartial^3^. It would be interesting for future research to explore how ex ante judgments of involved agents deviate from those of spectators. Despite the fact that spectators, just like involved agents, tend to take ex ante inequality into consideration^2,33^ and are also sensitive to the different sources causing inequality in outcomes^4,6,27^, previous studies have also pinpointed differences in the behavior of stakeholders and third-party individuals in general. To start with, spectators tend to fully achieve their distributive fairness ideals more often (due to this achievement being monetarily cheaper) than stakeholders^4,6^. Furthermore, when individuals are themselves affected by an unfair allocation of outcomes, they tend to punish fairness violations more strongly^51,52^ and show more intense emotional reactions to unfairness^53^ than third-party individuals do. Neural mechanisms in response to unfairness have also been reported to differ depending on whether individuals are directly affected by a fairness violation or a third-party observer in the situation^54,55^. Although these differences are restricted to the ex post domain, it is plausible that some differences will emerge between stakeholders and spectators in the ex ante domain, e.g., stakeholders are likely to act in more selfish ways. However, it is unclear whether there will be differences between stakeholders and spectators in how they evaluate the relative fairness of different factors generating unequal chances.

An additional interesting question for future research concerns the extent to which differences between cultures arise regarding ex ante fairness evaluation of chance inequality. It is well known in economics that different cultures exhibit different social preferences^56^ and it has been shown that ex post fairness views can differ substantially across cultures^27,57^. We can therefore also expect that ex ante fairness is affected by culture.

We point out that a limitation of our study is that participants always rated the source of inequality first in terms of fairness and then in terms of individual control. We chose this ordering because we are primarily interested in ex ante fairness evaluations, and only interested in control ratings to the extent that they relate to fairness. Considering the strength of the relationship between fairness and individual control and the fact that it is at the core of liberal egalitarianism^12^, our conjecture is that the positive correlation between fairness and control would not change substantially if we were to reverse the order of presentation between fairness and control. Nevertheless, we cannot fully rule out that asking participants first for their fairness or control ratings would not influence their assessments.

Finally, we realize that while in our study participants were clearly aware of the reason behind inequality in chances, this is often not the case in real life. Individuals’ misconceptions of the reasons why someone might have higher or lower chances might considerably affect their fairness judgments, and consequently their support for redistributive policies. Recent studies have found that ambiguity in the perception of others’ responsibility in their life outcomes leads to lower allocation of resources to them^58^, and that individuals are less supportive of income redistribution when it is unclear whether outcome inequality comes from effort or luck than when it clearly arises from luck^59^. It would be interesting to measure the contribution of these misconceptions to the opposition to and support for public policies that aim to establish ex ante equality, such as Affirmative Action.

## Supplementary information

Supplementary Information.

## Data Availability

The dataset generated and analysed during the current study is available from the authors on reasonable request.
