# Supplementary material for: Unequal chances: ex ante fairness and individual control
Source: Sci Rep. 2020 Dec 14;10:21862. doi: 10.1038/s41598-020-78335-w (PMC7736844; doi:10.1038/s41598-020-78335-w)
Supplement: Supplementary file 1 — Supplementary Information. [file 41598_2020_78335_MOESM1_ESM.pdf]

Unequal chances: Ex ante fairness and individual control

Leticia Micheli, Nickolas Gagnon

Supplementary information

Instructions Fairness

Consider a situation in which lottery tickets are distributed between two persons. Generally we ask you to assume that these two persons have a similar social, economic and educational background. In some cases this will not be so and you will be explicitly informed about it. Each ticket gives a 10% chance to win the lottery. There can be only one winner in the lottery, who receives 10 EUR as a prize. The ticket allocation is as follows: one person gets 9 tickets and thus has a 90% chance of winning the lottery prize, whereas the other person gets 1 ticket and thus has a 10% chance of winning the lottery prize. The allocation of tickets is based on various procedures. We would like to know how fair or unfair you consider each of the following procedures to be. Please also note that you cannot win the lottery prize in question.

Questions Fairness—Regular Block

Note for the reader: the titles of each question—displayed in Italics—were not shown to participants.

How fair/unfair would you consider the distribution of chances to win the lottery prize based on the following procedure:

-*Knowledge:* Chances are allocated based on the results in a general knowledge quiz. The person with the higher results receives a 90% chance to win, and the person with the lower results receives a 10% chance to win.

-*Effort:* Chances are allocated based on the results of a task that depends on effort. In the task, people see dots appearing randomly on horizontal bars on their computer screen. Their task is to position as many dots as possible in the middle of the horizontal bars. The person who places more dots in the correct position in a given time receives a 90% chance to win, and the person who places less dots in the correct position receives a 10% chance to win.

-*Luck:* Chances are allocated by throwing a 6-faced die. If the die number is smaller than or equal to 3, one person receives a 90% chance to win, and the other person receives a 10% chance to win. If the die number is greater than 3, the chances are reversed.

-*Height:* Chances are allocated based on height. The taller person receives a 90% chance to win, and the shorter person receives a 10% chance to win.

-*Weight:* Chances are allocated based on weight. The heavier person receives a 90% chance to win, and the lighter person receives a 10% chance to win.

-*Gender:* Chances are allocated based on gender. The two persons have different genders. The man receives a 90% chance to win, and the woman receives a 10% chance to win.

-*ID:* Chances are allocated based on student ID number. The person with the higher student ID number receives a 90% chance to win, and the person with the lower student ID number receives a 10% chance to win.

-*Nationality:* Chances between students at the School of Business and Economics of Maastricht University are allocated based on one's country of origin. The German student receives a 90% chance to win, whereas the non-German student receives a 10% chance to win.

-*Ethnicity:* Chances are allocated based on one's skin color. The two persons have different skin colors. The person with the lighter skin color receives a 90% chance to win, whereas the person with the darker skin color receives a 10% chance to win.

-*Benevolence:* Chances are allocated based on one's decision to help. The two persons both had an opportunity to help someone in need and only one decided to help. The person who helped receives a 90% chance to win, whereas the person who did not help receives a 10% chance to win.

-*Family Income:* Chances are allocated based on one's parental income. The person whose parents have a higher income receives a 90% chance to win, whereas the person whose parents have a lower income receives a 10% chance to win.

Instructions Individual Control

Consider the same situation in which lottery tickets are distributed between two persons. Generally we ask you to assume that these two persons have a similar social, economic and educational background. In some cases this will not be so and you will be explicitly informed about it. The allocation of tickets is based on various procedures. Now we would like to know how much control you consider people have over the distribution of chances for each of the following procedures. Again, there are no right or wrong answers. We are solely interested in your preferences and opinions. It is important for our research that you answer all questions honestly.

Questions Individual Control—Regular Block

How much control do you think people have over the chances they receive to win the lottery prize, when the distribution of chances is based on the following procedure.

Note for the reader: Factors were described in the same manner as in the *Fairness Instructions.*

Additional Information Concerning the Reverse Block

Although we did not present Luck as a factor in the Reverse Block, we present fairness and control ratings for Luck (obtained in the Regular Block) in the following tables for comparison purposes.

Factor	Luck	Family Income	Effort	ID	Knowledge	Benevolence	Nationality	Height	Ethnicity	Weight	Gender
Luck	4.63 (1.77)										
Family Income	***	2.74 (1.79)									
Effort	***		2.52 (1.65)								
ID	***			2.33 (1.66)							
Knowledge	***				2.17 (1.39)						
Benevolence	***		**			1.93 (1.44)					
Nationality	***	***	**	*	+		1.66 (1.21)				
Height	***	***	***	**	**			1.50 (.92)			
Ethnicity	***	***	***	**	**				1.44 (.98)		
Weight	***	***	***	***	***					1.35 (.62)	
Gender	***	***	***	***	***	*					1.32 (.83)

Notes: Standard deviations are indicated in parentheses. Stars in a cell indicate that the factor in the column is fairer than the factor in the corresponding row. Holm-Bonferroni-corrected p-values: + *p<.10* * *p<.05*; ** *p<.01*; *** *p<.001*

Table 1. Mean Fairness and Wilcoxon Signed-Rank Test Comparisons—Reverse Block

Factor	Benevolence	Effort	Knowledge	Weight	Luck	ID	Family Income	Height	Gender	Ethnicity	Nationality
Benevolence	5.61 (1.85)										
Effort	***	4.90 (1.70)									
Knowledge	***		4.63 (1.70)								
Weight	***	***	***	2.78 (1.66)							
Luck	***	***	***	***	1.55 (1.00)						
ID	***	***	***	***		1.46 (.88)					
Family Income	***	***	***	***			1.32 (.68)				
Height	***	***	***	***				1.29 (.76)			
Gender	***	***	***	***	+				1.23 (.73)		
Ethnicity	***	***	***	***						1.23 (.65)	
Nationality	***	***	***	***	**	*					1.20 (.74)

Notes: Standard deviations are indicated in parentheses. Stars in a cell indicate that the factor in the column is is under more control than the factor in the corresponding row. Holm-Bonferroni-corrected p-values: + *p<.10* * *p<.05*; ** *p<.01*; *** *p<.001*

Table 2. Mean Control and Wilcoxon Signed-Rank Test Comparisons—Reverse Block
